# Supplementary figures and images for: The AIRE G228W mutation disturbs the interaction of AIRE with its partner molecule SIRT1
Source: Front Immunol. 2022 Sep 6;13:948419. doi: 10.3389/fimmu.2022.948419 (PMC9485725; doi:10.3389/fimmu.2022.948419)

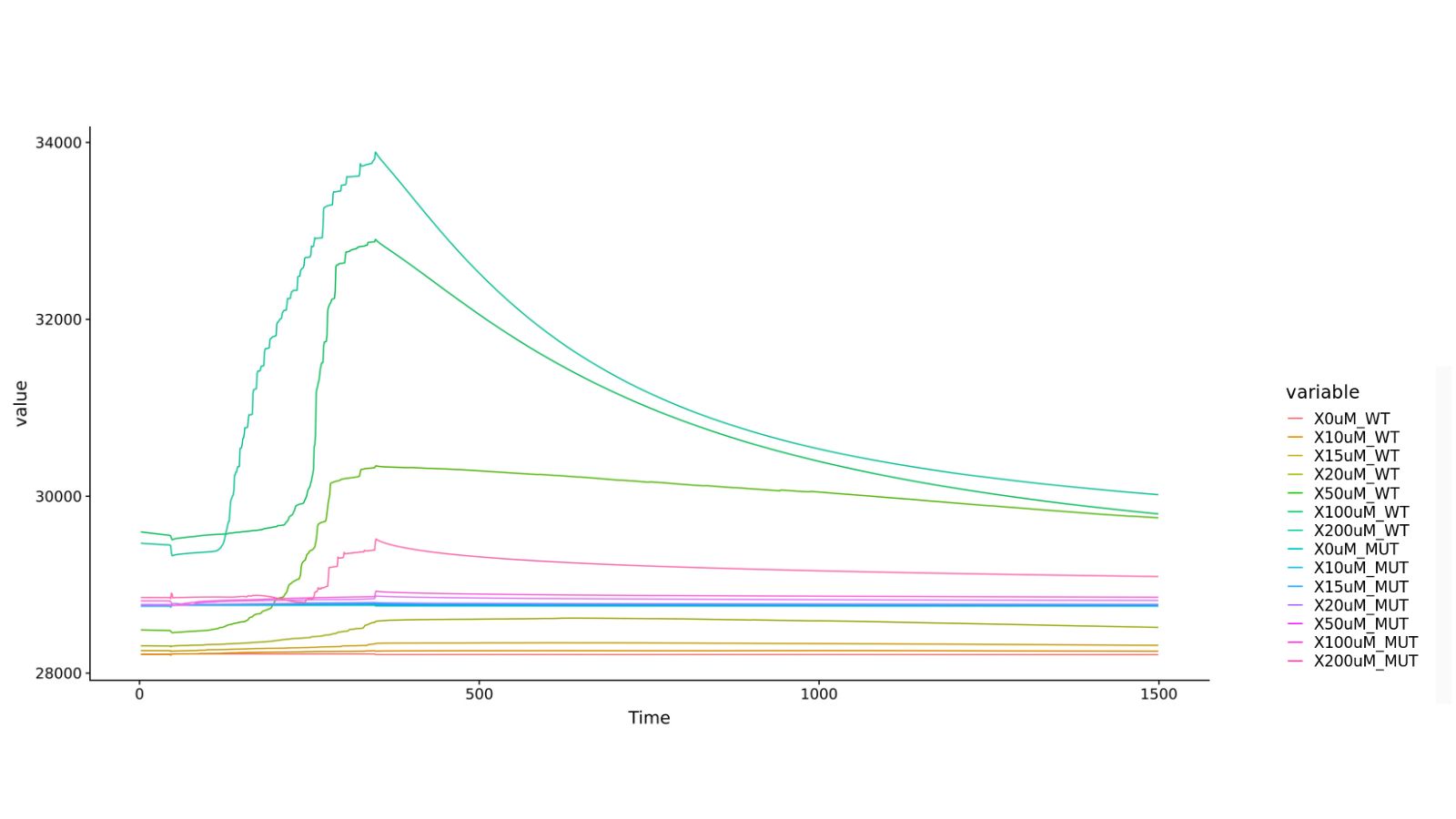

Supplement: Supplementary Figure 1 — Surface plasmon resonance sensorgram analysis of the binding between WT or G228W mutant AIRE SAND domain peptides with SIRT1 protein immobilized onto a Biacore sensor chip. Six different peptide concentrations were assayed (range 10, 15, 20, 50, 100, and 200 µM) plus the control (0 µM) for both WT and mutant peptides. Binding analysis shows a progressive increase in the association with the WT peptide, which was not observed with the mutant peptide. [file Image_1.jpeg]
